# The 2′-de­oxy­ribo­furan­oside of 3-phenyl­tetra­hydropyrimido[4,5-*c*]pyridazin-7-one: a bicyclic nucleoside with sugar residues in *N* and *S* con­formations, and its mol­ecular recognition

**DOI:** 10.1107/S2053229622005964

**Published:** 2022-06-13

**Authors:** Hui Mei, Simone Budow-Busse, Dasharath Kondhare, Henning Eickmeier, Hans Reuter, Frank Seela

**Affiliations:** a Laboratory of Bioorganic Chemistry and Chemical Biology, Center for Nanotechnology, Heisenbergstrasse 11, 48149 Münster, Germany; b Anorganische Chemie II, Institut für Chemie neuer Materialien, Universität Osnabrück, Barbarastrasse 7, 49069 Osnabrück, Germany; c Laboratorium für Organische und Bioorganische Chemie, Institut für Chemie neuer Materialien, Universität Osnabrück, Barbarastrasse 7, 49069 Osnabrück, Germany

**Keywords:** 2′-de­oxy­ribonucleoside, pyrimido[4,5-*c*]pyridazine, p*K*_a_ value, crystal structure, hydrogen bonding, base pair

## Abstract

The title compound 3-phenyltetra­hydro­pyrimido[4,5-*c*]pyridazine 2′-de­oxy­ribo­nucleoside [systematic name: 6-(2-deoxy-β-d-*erythro*-pentofuranosyl)-5,6,7,8-tetra­hydro-3-phenylpyrimido[4,5-*c*]pyridazin-7-one monohydrate, C_17_H_18_N_4_O_4_·H_2_O, **1**] shows two con­formations in the crystalline state and the two con­formers (**1a** and **1b**) adopt different sugar puckers. The sugar residue of **1a** shows a C2′-*endo S*-type con­formation, while **1b** displays a C3′-*endo N*-type sugar pucker. Both con­formers adopt similar *anti* con­formations around the N-glycosylic bonds, with χ = −97.5 (3)° for con­former **1a** and χ = −103.8 (3)° for con­former **1b**. The extended crystalline network is stabilized by several inter­molecular hydrogen bonds involving nucleoside and water mol­ecules. The nucleobases and phenyl substituents of the two con­formers (**1a** and **1b**) are stacked and display a reverse alignment. A Hirshfeld surface analysis supports the hydrogen-bonding pattern, while curvedness surfaces visualize the stacking inter­actions of neighbouring mol­ecules. The recognition face of nucleoside **1** for base-pair formation mimics that of 2′-de­oxy­thymidine. Nucleoside **1** shows two p*K*_a_ values: 1.8 for pro­ton­ation and 11.2 for deprotonation. DNA oligonucleotides containing nucleoside **1** were synthesized and hybridized with com­plementary DNA strands. Nucleoside **1** forms a stable base pair with dA which is as stable as the canonical dA–dT pair. The bidentate **1**–dA base pair is strengthened by a third hydrogen bond provided by the dA analogue 3-bromo­pyrazolo­[3,4-*d*]pyrimidine-4,6-di­amine 2′-de­oxy­ribo­furan­oside (**4**). By this, duplex stability is increased and the sug­gested base-pairing patterns are supported.

## Abstract 2

3-Phenyl­tetra­hydro­pyrimido[4,5-*c*]pyridazine 2′-de­oxy­ribonucleoside shows two con­formations in the crystalline state with distinct differences observed for the sugar moiety (*N versus S* sugar pucker). The nucleoside mimics the recognition face of dT and forms stable base pairs with dA.

## Introduction

Nucleosides with artificial nucleobases offer new functionalities not existing in the canonical constituents of DNA and RNA. Alteration of the nitro­gen pattern and functionalization with additional substituents are methods to change mol­ecular recognition and base-pair stability. Artificial nucleosides were used to probe inter­actions in DNA and RNA, or with proteins and other biomolecules. In addition, DNA is utilized in materials science for information storage or as a nanomaterial (Meiser *et al.*, 2020 ▸). Entirely new base pairs were constructed to expand the repertoire of nucleic acid applications (Hirao *et al.*, 2012 ▸). Often, only minor structural changes are needed to achieve these objectives.

Pyrimido[4,5-*c*]pyridazine 2′-de­oxy­ribonucleoside **1** [Scheme 1 shows nucleoside **1** and structurally related com­pounds, with the recognition sites according to dT (red) and dC (blue)] can serve as a mimic for 2′-de­oxy­thymidine (dT). Nucleoside **1** shows ambiguous base-pair recognition (Topal & Fresco, 1976 ▸). Nevertheless, it is able to distinguish between canonical purine and pyrimidine nucleosides with a preference for com­plementary 2′-de­oxy­adenosine (dA) (Mei *et al.*, 2015 ▸). This is contrary to the related nucleosides **2** and **3**, which display the recognition face of 2′-de­oxy­cytidine (dC). Com­pound **1** has a strong structural relationship to pyrrolo-dC (**2**) and imidazolo-dC (**3**) (see Scheme 1), and is decorated with a phenyl ring, as is the case for **2** and **3** (Hudson & Ghorbani-Choghamarani, 2007 ▸; Mei *et al.*, 2014 ▸). A 3-methyl­pyrimido[4,5-*c*]pyridazine nucleo­side (Loakes *et al.*, 2003*a* ▸,*b* ▸) and a phenyl­ethyl derivative were reported previously (Mieczkowski *et al.*, 2016 ▸). Nucleoside **1** was synthesized in our laboratory and incorporated into DNA oligonucleotides employing phospho­ramidite chemistry and solid-phase oligonucleotide synthesis (Mei *et al.*, 2015 ▸). Chemical synthesis of DNA oligonucleotides has several advantages over triphos­phate incorporation catalyzed by polymerases, especially when modified nucleosides are used. Modified nucleosides are often not sufficiently accepted by DNA polymerases and therefore chain elongation succeeds only in low yields or is terminated (Hollenstein, 2012 ▸). DNA oligonucleotide synthesis with nucleoside phospho­ramidites can be performed even with highly modified nucleosides and is a standard method in the field of nucleic chemistry. 
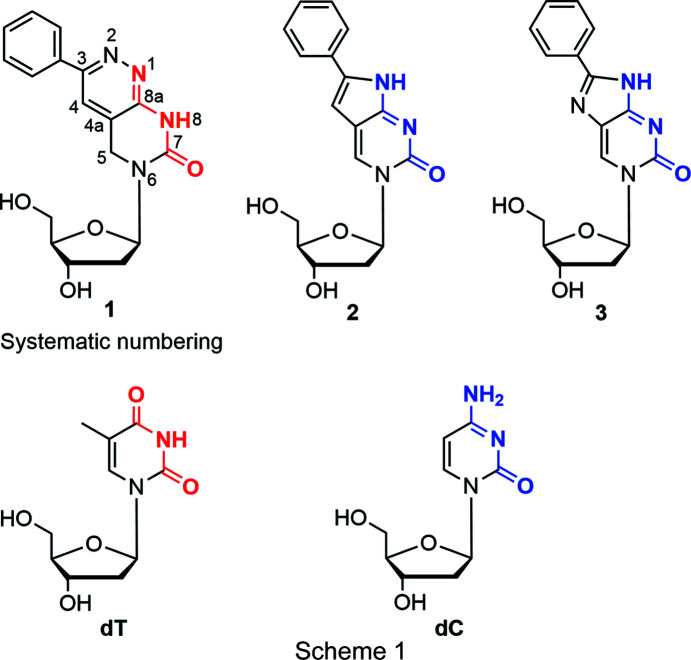


To obtain detailed information on its con­formation and crystal packing in the solid state, a single-crystal X-ray analysis of nucleoside **1** was performed. A Hirshfeld surface analysis was carried out to visualize the packing inter­actions. DNA oligonucleotides containing phenyl­pyrimido[4,5-*c*]pyridazine nucleoside **1** were synthesized and hybridization experiments were performed to strengthen the bidentate **1**–dA base pair (Mei *et al.*, 2015 ▸) by replacement with a tridentate base pair employing 3-bromo­pyrazolo­[3,4-*d*]pyrimidine-4,6-di­amine 2′-de­oxy­ribo­furan­oside, **4** (Seela & Becher, 2001 ▸; He *et al.*, 2003 ▸), as dA surrogate.

## Experimental

### Synthesis and crystallization of 1

Nucleoside **1** was synthesized as described previously (Mei *et al.*, 2015 ▸). Colourless crystals of **1** were obtained from a hot methanol/water mixture (m.p. 450 K). For the X-ray crystallographic analysis, a single crystal was mounted on a MiTeGen Micro-Mounts fibre in a thin smear of oil.

### Refinement

Crystal data, data collection and structure refinement details are summarized in Table 1 ▸. The known configuration of the parent mol­ecule was used to define the enanti­omer employed in the refined model. In the absence of suitable anomalous scattering, Friedel equivalents could not be used to determine the absolute structure. Refinement of the Flack (1983 ▸) parameter led to inconclusive values for this parameter [0.0 (6)]. All H atoms were found in a difference Fourier synthesis. In order to maximize the data/parameter ratio, the H atoms were placed in geometrically idealized positions, with C—H = 0.95–1.00 Å, and were constrained to ride on their parent atoms, with *U*_iso_(H) = 1.2*U*_eq_(C) = *U*_eq_(N). The hy­droxy groups were refined as groups allowed to rotate but not tip, with O—H = 0.84 Å and *U*_iso_(H) = 1.5*U*_eq_(O).

## Results and discussion

### Mol­ecular geometry and con­formation of 1

**Figure 1:**
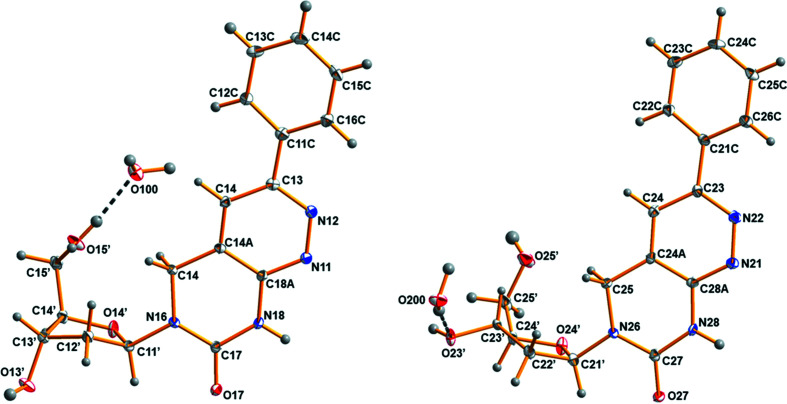
Perspective views and the atom-numbering schemes of con­formers **1a** and **1b**, each forming a hydrogen bond to a water mol­ecule (dashed line). Displacement ellipsoids are drawn at the 50% probability level.

The crystals of phenyl­pyrimido[4,5-*c*]pyridazine nucleoside **1** are ortho­rhom­bic with the space group *P*2_1_2_1_2_1_. There are two mol­ecules of **1** in the asymmetric unit, denoted as con­former **1a** and con­former **1b**. As shown in Fig. 1 ▸, each con­former is connected to a water mol­ecule *via* hydrogen bonding. Selected geometric parameters are summarized in Table 2 ▸.

The orientation of the nucleobase relative to the sugar residue (*syn*–*anti*) is defined by the torsion angle χ(O4′—C1′—N9—C4) (IUPAC–IUB Joint Commission on Biochemical Nomenclature, 1983 ▸), and the preferred con­formation around the N-glycosidic bond is *anti* for canonical purine 2′-de­oxy­ribonucleosides (Saenger, 1984 ▸). For pyrimido[4,5-*c*]pyridazine nucleoside **1**, the torsion angle χ(O4′—C1′—N6—C7) is defined in analogy to natural nucleosides, as this mol­ecule can be considered as a purine nucleoside analogue. Both con­formers of mol­ecule **1** adopt similar *anti* con­formations, with χ = −97.5 (3)° for con­former **1a** and χ = −103.8 (3)° for con­former **1b**.

The pyridazine rings of **1a** and **1b** are nearly planar. For **1a**, the deviations of the ring atoms (N11/N12/C13/C14/C14*A*/C18*A*) from the least-squares plane range from 0.013 (2) Å for atom C14*A* to −0.013 (2) Å for atom C18*A*, with an r.m.s. deviation of 0.0091 Å. In the case of con­former **1b**, the r.m.s. deviation of the ring atoms from their calculated least-squares planes is 0.0216 Å and the range is from 0.032 (2) Å for atom C28*A* to −0.022 (2) Å for atom C24*A*. The presence of the *sp* ^3^-hybridized C15/C25 atom causes a displacement of the C atom from the mean plane in both con­formers com­pared to a reduced pyrimidine moiety. In **1a**, atom C15 is displaced by 0.081 (4) Å from the mean plane, while for **1b** the displacement of atom C25 is −0.134 (4) Å. The corresponding N6—C5—C4*A* bond angle is 112.7 (2)° for **1a** and 112.8 (2)° for **1b**.

In both con­formers, the pyridazine ring and the phenyl substituent are slightly tilted with respect to each other, with C16*C*—C11*C*—C13—N12 = −16.1 (4)° for **1a** and C26*C*—C21*C*—C23—N22 = −6.8 (4)° for **1b**. The C3—C1*C* bond connecting the phenyl moiety with the pyridazine ring is almost identical for both con­formers [C13—C11*C* = 1.483 (4) Å for **1a** and C23—C21*C* = 1.486 (4) Å for **1b**]. Also, the N6—C1′ bond connecting the nucleobase and the sugar moiety is of com­parable length [1.457 (3) Å for **1a** and 1.448 (3) Å for **1b**].

The most pronounced differences between con­formers **1a** and **1b** concern the con­formation of the sugar moiety. The sugar moiety of nucleosides can adopt two principal puckering modes, namely, C3′-*endo* (*N*) and C2′-*endo* (*S*), corresponding to the major dislocation of C3′ or C2′ from the median plane of C1′—O4′—C4′ (Altona & Sundaralingam, 1972 ▸; Saenger, 1984 ▸). For canonical 2′-de­oxy­ribonucleosides, the preferred sugar con­formation is C2′-*endo*. Moreover, the torsion angle γ(O5′—C5′—C4′—C3′) characterizes the orientation of the exocyclic 5′-hy­droxy group relative to the sugar moiety (Saenger, 1984 ▸). The 2′-de­oxy­ribose ring of **1a** also adopts a C2′-*endo S*-type con­formation (C3′-*exo*–C2′-*endo*, _3_ *T* ^2^), with a pseudorotational phase angle *P* = 182.7 (2)° and a maximum amplitude τ_*m*_ = 33.9 (1)°. The con­formation about the C4′—C5′ bond is anti­periplanar (+*ap*), with the torsion angle γ = 177.2 (2)°. In contrast, a C3′-*endo N*-type (C3′-*endo*–C4′-*exo*, ^3^ *T*_4_) sugar con­formation is observed for **1b**, with *P* = 34.6 (2)° and τ_*m*_ = 32.4 (1)°. The 5′-hy­droxy group of con­former **1b** adopts a synclinal (+*sc*) con­formation, with γ = 54.4 (3)°.

The con­formational differences of con­formers **1a** and **1b**, which mainly concern the sugar moiety (*N versus S* con­formation), are probably the consequence of the different hydrogen-bonding sites of the sugar residues to nearby water mol­ecules.

### Hydrogen bonding and mol­ecular packing of 1

**Figure 2:**
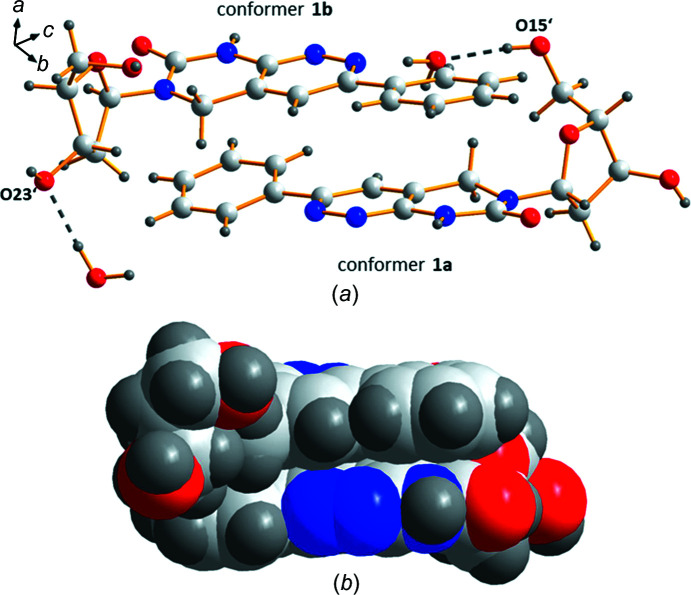
(*a*) Reverse alignment of con­formers **1a** and **1b**, and hydrogen bonding to water mol­ecules (dashed lines). (*b*) Space-filling model of a com­pact unit consisting of con­formers **1a** and **1b**.

The crystalline structure of phenyl­pyrimido[4,5-*c*]pyrida­zine nucleoside **1** is stabilized by a heterogeneous network consisting of several inter­molecular hydrogen bonds which involve the nucleoside and water mol­ecules (Table 3 ▸). The hydrogen bonds formed by the water mol­ecules are particularly important as they stabilize the different sugar con­formations of the two con­formers (**1a** and **1b**). Notably, con­former **1a** with a C2′-*endo* (*S*) con­formation forms a hydrogen bond to nearby water mol­ecules only *via* its 5′-hy­droxy group, while the sugar moiety of con­former **1b**, with a C3′-*endo* (*N*) con­formation, has multiple contacts to nearby water mol­ecules. This includes the 3′- and 5′-hy­droxy groups, and atom O24′ of the furan­ose ring [for details and symmetry codes, see Table 3 ▸ and Fig. 2 ▸(*a*)].

Conformers **1a** and **1b** are stacked with a reverse alignment with respect to each other, forming a com­pact unit. Within this unit, hydrogen-bond formation is not observed between the con­formers. As shown in Figs. 2 ▸(*a*) and 2(*b*), the phenyl sub­stituent of each con­former faces the pyrimidine ring of the nucleobase of the other con­former. In addition, each sugar residue points towards the other con­former. This is somewhat different to the arrangement in the crystal structure of the closely related 3-methyl­pyrimido[4,5-*c*]pyridazine nucleoside (Loakes *et al.*, 2003*a* ▸), wherein the sugar units point away from the other con­former (see Fig. S1 in the supporting information).

**Figure 3:**
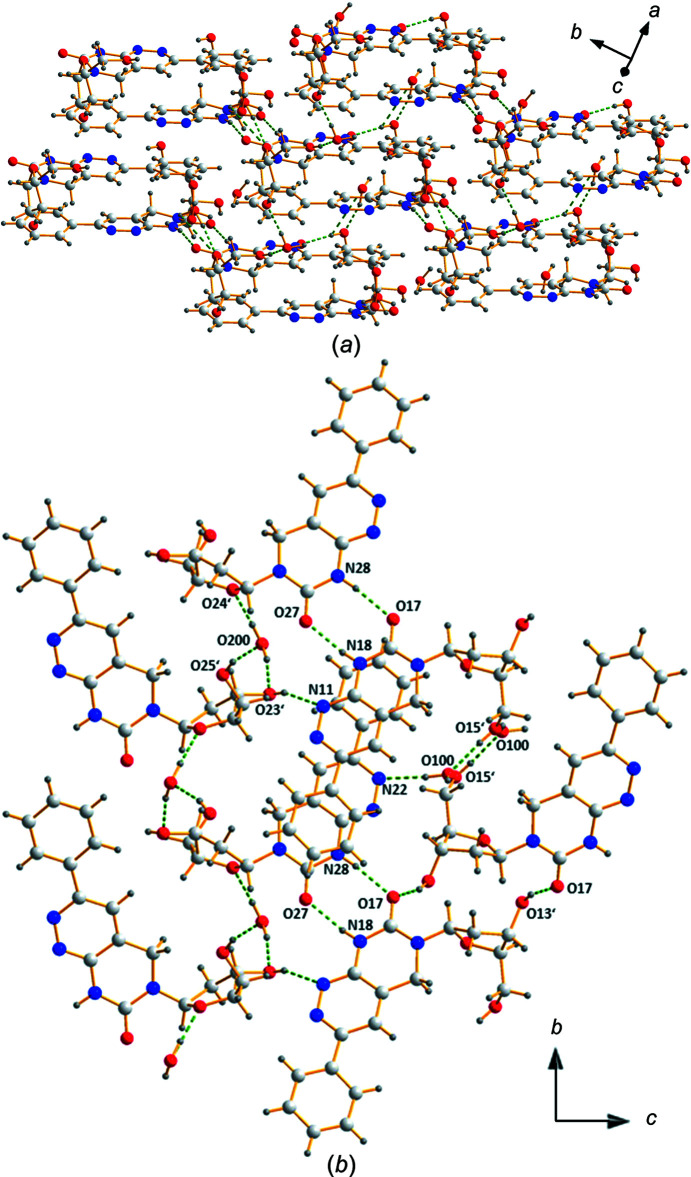
(*a*) Packing of con­formers (**1a** and **1b**) within the extended crystalline network. (*b*) Detailed view of the hydrogen-bonding scheme (dashed lines), shown parallel to the *bc* plane.

The arrangement of con­formers **1a** and **1b** within the extended crystalline network and the hydrogen-bonding scheme is shown in Fig. 3 ▸ and the supporting information (Fig. S2). The two con­formers are linked by hydrogen bonds formed between neighbouring pyrimidine moieties of the other con­former with atom N8 as donor and atom O7 as acceptor (N18—H18N⋯O27^i^ and N28—H28N⋯O17^iv^). Most inter­estingly, atom O17 of con­former **1a** also functions as an acceptor for a hydrogen bond with the 3′-hy­droxy group of another mol­ecule of **1a** (O13′—H13O⋯O17^ii^), while this kind of contact is not observed for con­former **1b**. On the other hand, pyridazine atom N22 is the acceptor for a contact to a nearby water mol­ecule (O100—H100⋯N22), whereas the corresponding atom N12 of con­former **1a** is not involved in hydrogen bonding.

In addition, the arrangement of the nucleobases results in π–π stacking between the phenyl and pyrimido[4,5-*c*]pyrida­zine rings, as shown in Fig. 3 ▸(*a*), with inter­atomic distances ranging from 3.12 (N12⋯C24) to 3.69 Å (N16⋯C25*C*). The π–π inter­action of the ring systems is supported by the Hirshfeld surface analysis of nucleoside **1** (see next section).

### Hirshfeld surface analysis of nucleoside 1

**Figure 4:**
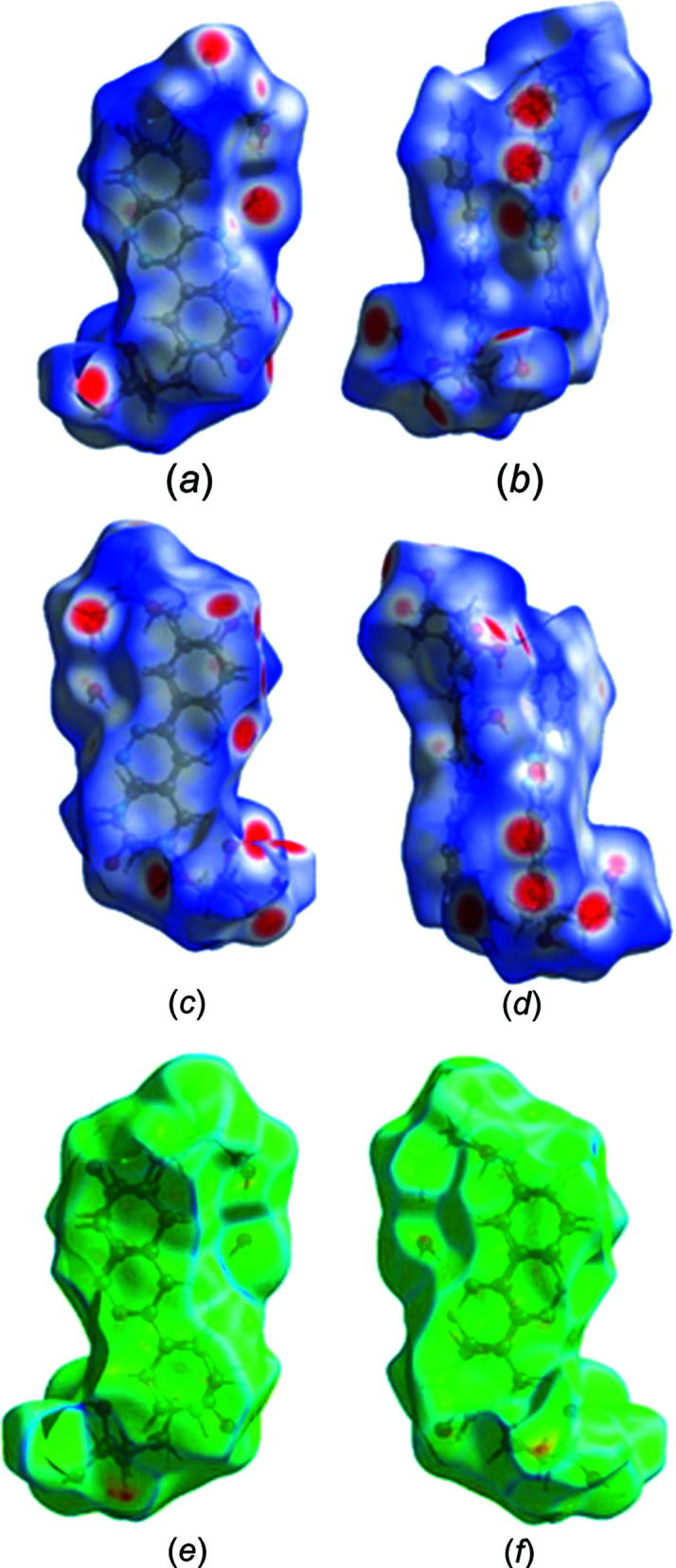
Hirshfeld surfaces of con­formers **1a** and **1b** mapped with *d*_norm_ (0.5 to 1.5 Å), shown in (*a*) front, (*b*)/(*c*) side and (*d*) back views. The curvedness surfaces of the two con­formers of nucleosides **1a** and **1b** are shown in (*e*) front and (*f*) back views.

The Hirshfeld surface analysis, including three-dimensional (3D) surfaces and two-dimensional (2D) fingerprint plots, provides additional insight into the role of crystal packing forces and visualizes the relative strengths of inter­molecular inter­actions of crystalline com­pounds. The program *CrystalExplorer* (Version 17; Spackman & Jayatilaka, 2009 ▸; Turner *et al.*, 2017 ▸) was used to carry out a Hirshfeld surface analysis of phenyl­pyrimido[4,5-*c*]pyridazine nucleoside **1**, mapped in the *d*_norm_ range from −0.5 to 1.5 Å, shape index (−1.0 to 1.0 Å) (see Fig. S3 in the supporting information) and curvedness (−4.0 to 0.4 Å), as well as a 2D fingerprint plot analysis. The Hirshfeld surfaces depicted in Figs. 4 ▸(*a*)–(*d*) show several deep-red spots representing short contacts, while white surface areas indicate contacts with distances equal to the sum of the van der Waals radii. The red spots correspond to the close O—H⋯O and N—H⋯O contacts of the mol­ecules and confirm the hydrogen-bonding data (Table 3 ▸). In addition, the curvedness surfaces show a large and relatively flat green region covering the pyrimido[4,5-*c*]pyridazine nucleobase and the phenyl substituent [Figs. 4 ▸(*e*) and 4(*f*)]. This indicates the presence of π–π stacking inter­actions with neighbouring mol­ecules and fits the crystal packing scheme wherein the heterocyclic nucleobases and the phenyl substituent of the two con­formers (**1a** and **1b**) face each other with a reverse orientation [Fig. 2 ▸(*a*)].

**Figure 5:**
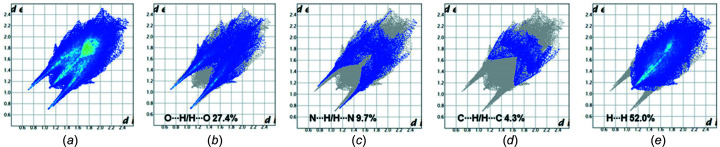
2D fingerprint plots showing the percentage contributions of various inter­actions to the total Hirshfeld surface area of the two con­formers of com­pound **1**: (*a*) full inter­actions and resolved contacts; (*b*) O⋯H/H⋯O; (*c*) N⋯H/H⋯N; (*d*) C⋯H/H⋯C; (*e*) H⋯H.

Fig. 5 ▸ shows the overall 2D fingerprint plot of mol­ecule **1** [Fig. 5 ▸(*a*)] and the plots resolved into O⋯H/H⋯O, N⋯H/H⋯N, C⋯H/H⋯C and H⋯H contacts [Figs. 5 ▸(*b*)–(*e*)] to highlight the particular atom-pair inter­actions, together with their relative contributions to the Hirshfeld surface. The proportions of O⋯H/H⋯O and N⋯H/H⋯N inter­actions com­prise 27.4 and 9.7%, respectively, of the total Hirshfeld surfaces. The H⋯H and C⋯H/H⋯C contacts amount to 52.0 and 4.3%, respectively, and suggest that van der Waals inter­actions also play a role in the crystal packing of nucleoside **1**.

### p*K* values and base pairing

**Figure 6:**
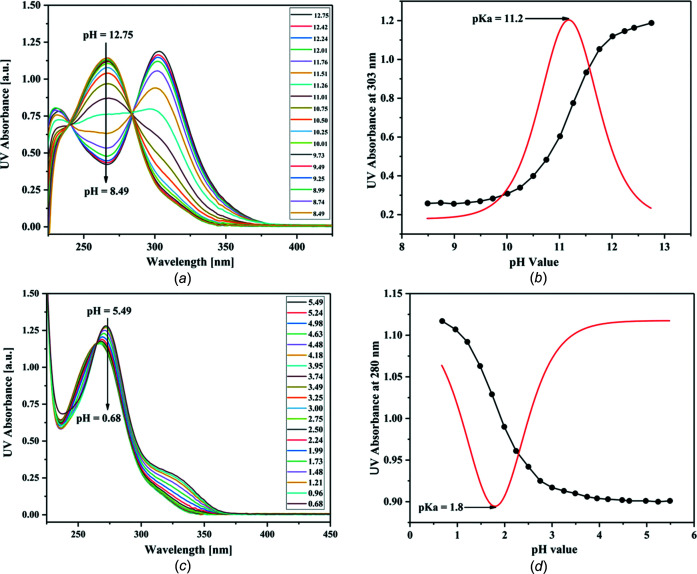
(*a*) pH-dependent UV spectra of **1** measured in phosphate buffer from pH 12.75 to pH 8.49. (*b*) Absorbance of **1** at 303 nm *versus* pH value and its first derivative using data from part (*a*). (*c*) pH-dependent UV spectra of **1** measured in phosphate buffer from pH 5.49 to pH 0.68. (*d*) Absorbance of **1** at 280 nm *versus* pH value and its first derivative using data from part (*c*).

**Figure 7:**
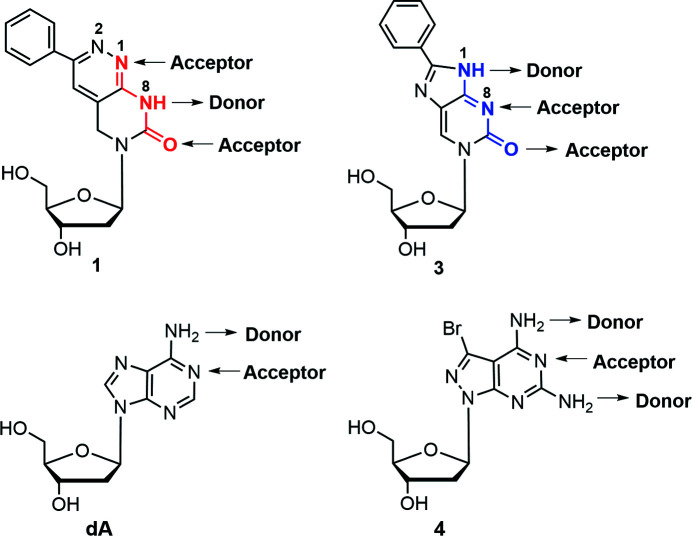
Donor–acceptor pattern of nucleosides **1**, **3**, dA and **4**.

**Figure 8:**
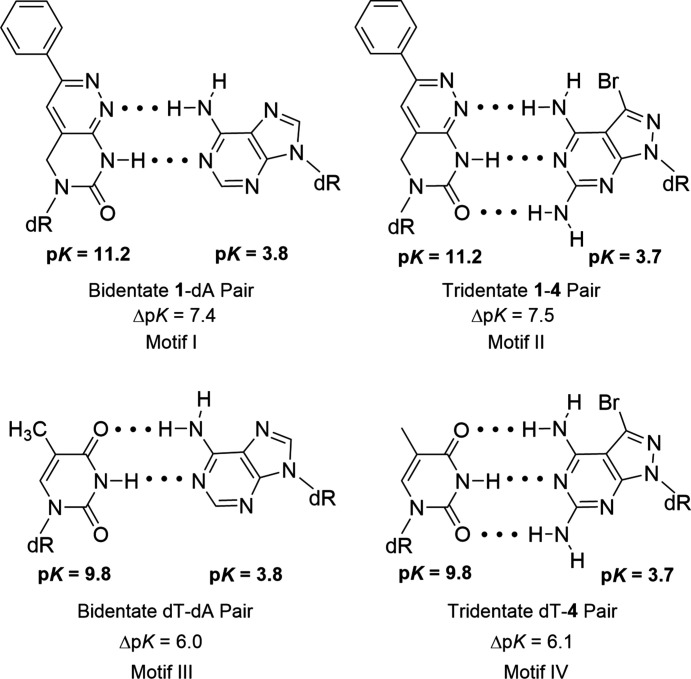
Base-pairing motifs, p*K* values of base-pairing nucleosides and their p*K* value differences (Δp*K*).

The p*K*_a_ values (ionization or dissociation constants) of canonical and modified nucleobases are an important parameter for the prediction of base-pairing properties in terms of their lifetime and stability. Accordingly, the p*K* value of nucleoside **1** was determined and com­pared to that of dT. For p*K* determination, the spectrophotometric UV titration of **1** was performed and the dependency of a continuously increased pH value and absorption data were plotted against pH values. To cover the full range of the pH scale, measurements were carried out between pH 12.8 and pH 8.5 [Fig. 6 ▸(*a*)], as well as between pH 5.5 and pH 0.7 [Fig. 6 ▸(*c*)]. Due to the two-state equilibrium of the protonated and deproton­ated species, isosbestic points are observed in the UV spectra [Figs. 6 ▸(*a*) and 6(*c*)]. Fig. 6 ▸(*b*) displays a p*K* value of 11.2 for the deprotonation of nucleoside **1**. This is higher than that of dT (9.8) and makes the deprotonation of **1** more difficult. The p*K*_a_ value of protonation of **1** was found to be 1.8 [Fig. 6 ▸(*d*)]. dT has no p*K* value in this range. For the protonation of **1**, nitro­gen-1 and nitro­gen-3 are the possible proton-acceptor sites (Fig. 7 ▸). Earlier, it was reported that strong base pairs are formed when the p*K* value difference (Δp*K*) between the acceptor and donor sites of nucleobases is greater than 5 units (Krishnamurthy, 2012 ▸). Thus, p*K* value differences were calculated for the base-pair motifs shown in Fig. 8 ▸. Similar Δp*K* values were found for the **1**–dA and dT–dA base pairs, which supports stable base-pair formation. Base pairing of a related com­pound with a methyl group instead of the phenyl group was reported previously (Loakes *et al.*, 2003*a* ▸). However, base-pair motifs were not given and p*K* values were not determined.

The X-ray crystal structure of **1** reported in this study unambiguously shows that the H atom is located at nitro­gen-8 (pyrimidine ring; Fig. 1 ▸) and can act as proton donor for **1**–dA base pairing. This is different to nucleosides **2** and **3**, which carry the H atoms at nitro­gen-1 (imidazole/pyrrole ring). According to the shift of the proton-donor site from nitro­gen-1 to nitro­gen-8, N1 now becomes an acceptor site in nucleoside **1**. This is a consequence of the ring displacement (pyridazine instead of pyrrole or imidazole) and makes nucleoside **1** an analogue of dT, whereas com­pounds **2** and **3** are analogues of dC (Fig. 7 ▸).

The dA–dT base pair can be stabilized when an additional amino group is added at the 2-position of the adenine base (Chazin *et al.*, 1991 ▸). These stabilizers make use of the principle of third hydrogen-bond formation. 3-Bromo­pyra­zolo­[3,4-*d*]pyrimidin-4,6-di­amine 2′-de­oxy­ribo­furan­oside, **4**, has been used as a stabilizer for the dA–dT base pair (Seela & Becher, 2001 ▸; He *et al.*, 2003 ▸). Com­pared to dA, nucleoside **4** contains an additional amino group at position-2 that can participate in a third hydrogen bond with dT (Figs. 7 ▸ and 8 ▸). This causes a stabilization of the base pair and increases the thermal stability of DNA (Table 4 ▸).

We anti­cipated that a similar stabilization should take place when nucleoside **1** is part of the **1**–dA base pair. Accordingly, an increased tem­per­ature of duplex dissociation (*T*_m_) should be observed. The *T*_m_ value is a measure for the stability of a double-stranded DNA and depends on the stability of its base pairs. It is measured spectrophotometrically (UV) at 260 nm and can be followed by the absorbance change with increasing tem­per­ature and the transition from double- to single-stranded DNA.

To this end, DNA oligonucleotides ODN-**1** to ODN-**6** were synthesized and hybridization experiments were performed (for experimental details, see the supporting information). The *T*_m_ data are summarized in Table 4 ▸ and melting profiles are displayed in Fig. S4 in the supporting information. According to the *T*_m_ data, a stability increase is observed from 47 °C for the duplex containing the **1**–dA base pair to 50 °C (+3 °C) for the duplex ODN-**5**–ODN-**6** incorporating the **1**–**4** pair. Apparently, a tridentate **1**–**4** base pair is formed. Nevertheless, the increase induced by the **1**–**4** pair is lower than that for the **4**–dT pair (*T*_m_ = 54 °C; +7 °C). Obviously, the formation of the third hydrogen bond is less efficient in the **1**–**4** base pair than for the dT pair with **4**. Electronic and geometric properties of the nucleobases including altered stacking inter­actions might account for this behaviour. Possible base-pairing motifs for the **1**–dA and **1**–**4** pairs are displayed in Fig. 8 ▸, together with the motifs of the dT–dA and dT–**4** pairs.

## Conclusion

Phenyl­pyrimido[4,5-*c*]pyridazine 2′-de­oxy­ribonucleoside **1** forms two con­formers (**1a** and **1b**) in the solid state. Conformer **1a** displays a C2′-*endo S*-type sugar pucker, whereas con­former **1b** adopts a C3′-*endo N*-type con­formation. Both con­formers show *anti* con­formations around the N-glycosylic bonds, with χ = −97.5 (3)° for con­former **1a** and χ = −103.8 (3)° for con­former **1b**. The extended crystalline structure of nucleoside **1** is stabilized by a heterogeneous hydrogen-bond network involving the nucleoside and water mol­ecules. Conformers **1a** and **1b** are placed opposite each other with a reverse alignment. Strong stacking inter­actions are observed for the nucleobase and the phenyl ring decorating the heterocycle. A Hirshfeld surface analysis supports the hydrogen-bonding scheme, while the curvedness surfaces visualize the stacking inter­actions of neighbouring mol­ecules.

Nucleoside **1** mimics the recognition face of dT and is deprotonated under alkaline conditions (**1**: p*K*_a_ = 11.2; dT: p*K*_a_ = 9.8). DNA duplexes obtained by hybridization of com­plementary oligonucleotides form a stable **1**–dA base pair that is as stable as the canonical dA–dT pair. The stability of the **1**–dA base pair is increased when the oxo group of **1** participates in a third hydrogen bond. This is the case when dA in the dA–**1** base pair is replaced by the 2-amino stabilizer **4** which provides an additional amino group for tridentate base-pair formation.

## Supplementary Material

Crystal structure: contains datablock(s) I, global. DOI: 10.1107/S2053229622005964/cu3182sup1.cif

Structure factors: contains datablock(s) I. DOI: 10.1107/S2053229622005964/cu3182Isup2.hkl

Additional figures, shape index surfaces, oligonucleotide syntheses and characterization, melting curves. DOI: 10.1107/S2053229622005964/cu3182sup3.pdf

CCDC reference: 2176720

## Figures and Tables

**Table 1:** Experimental details

Crystal data	
Chemical formula	C_17_H_18_N_4_O_4_·H_2_O
*M*_r_	360.37
Crystal system, space group	Orthorhombic, *P*2_1_2_1_2_1_
Tem­per­ature (K)	100
*a*, *b*, *c* (Å)	7.2057 (3), 11.0792 (4), 41.2346 (16)
*V* (Å^3^)	3291.9 (2)
*Z*	8
Radiation type	Mo *K*α
μ (mm^−1^)	0.11
Crystal size (mm)	0.19 × 0.16 × 0.09

Data collection	
Diffractometer	Bruker APEXII CCD
Absorption correction	Multi-scan (*SADABS*; Bruker, 2008 ▸)
*T*_min_, *T*_max_	0.979, 0.991
No. of measured, independent and observed [ > *I* > 2σ(*I*)] reflections	91832, 4528, 3773
*R*_int_	0.118
(sin θ/λ)_max_ (Å^−1^)	0.661

Refinement	
*R*[*F* ^2^ > 2σ(*F* ^2^)], *wR*(*F* ^2^), *S*	0.045, 0.096, 1.07
No. of reflections	4528
No. of parameters	473
H-atom treatment	H atoms treated by a mixture of independent and constrained refinement
Δρ_max_, Δρ_min_ (e Å^−3^)	0.27, −0.31
Absolute structure	Established by known chemical absolute configuration

com­puter programs: *APEX2* (Bruker, 2008 ▸), *SAINT* (Bruker, 2008 ▸), *SHELXTL* (Sheldrick, 2008 ▸), *DIAMOND* (Brandenburg, 2005 ▸), *SHELXTL* (Sheldrick, 2008 ▸) and *PLATON* (Spek, 2020 ▸).

**Table 2:** Selected geometric parameters (Å, °)

C11*C*—C13	1.483 (4)	C21*C*—C23	1.486 (4)
N16—C11′	1.457 (3)	N26—C21′	1.448 (3)
			
N16—C15—C14*A*	112.7 (2)	N26—C25—C24*A*	112.8 (2)
			
C16*C*—C11*C*—C13—N12	−16.1 (4)	C26*C*—C21*C*—C23—N22	−6.8 (4)
C17—N16—C11′—O14′	−97.5 (3)	C27—N26—C21′—O24′	−103.8 (3)
C13′—C14′—C15′—O15′	177.20 (19)	C23′—C24′—C25′—O25′	54.4 (3)

**Table 3:** Hydrogen-bond geometry (Å, °)

*D*—H⋯*A*	*D*—H	H⋯*A*	*D*⋯*A*	*D*—H⋯*A*
N18—H18N⋯O27^i^	0.88	1.92	2.773 (3)	162
O13′—H13O⋯O17^ii^	0.84	2.15	2.968 (3)	166
O15′—H15O⋯O100	0.84	1.91	2.736 (2)	169
O100—H101⋯O15′^iii^	0.96	1.86	2.781 (3)	160
O100—H100⋯N22	0.96	1.99	2.946 (3)	175
N28—H28N⋯O17^iv^	0.88	1.95	2.823 (3)	173
O23′—H23O⋯N11^v^	0.84	2.05	2.846 (3)	158
O25′—H25O⋯O200^vi^	0.84	1.90	2.721 (3)	164
O200—H201⋯O24′^vii^	0.96	1.84	2.801 (3)	175
O200—H200⋯O23′	0.96	1.89	2.836 (3)	169

Symmetry codes: (i) 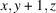 ; (ii) 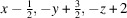 ; (iii) 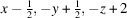 ; (iv) 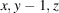 ; (v) 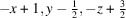 ; (vi) 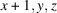 ; (vii) 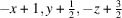 .

**Table 4:** *T*_m_ values of DNA oligonucleotide duplexes containing base pairs formed by nucleosides **1** and **4** ^*a*^

Duplex	*T*_m_ *^*b*^* (°C)
5′-d(TAG GTC AAT ACT) (ODN-**1**) 3′-d(ATC CAG TTA TGA) (ODN-**2**)	47
5′-d(TAG GTC 4AT ACT) (ODN-**3**) 3′-d(ATC CAG TTA TGA) (ODN-**2**)	54
5′-d(TAG GTC AAT ACT) (ODN-**1**) 3′-d(ATC CAG 1TA TGA) (ODN-**4**)	47
5′-d(TAG GTC 1AT ACT) (ODN-**5**) 3′-d(ATC CAG 4TA TGA) (ODN-**6**)	50

Notes: (*a*) measured at 260 nm at a concentration of 2 µ*M* + 2 µ*M* single strand at a heating rate of 1 °C min^−1^ in 100 m*M* NaCl, 10 m*M* MgCl_2_ and 10 m*M* Na cacodylate (pH 7.0). (*b*) *T*_m_ values were calculated from the heating curves using the program *Meltwin* (Version 3.0; McDowell & Turner, 1996 ▸) and are given with an error of ±5%.
