# Supplementary material for: The 2′-de­oxy­ribo­furan­oside of 3-phenyl­tetra­hydropyrimido[4,5-c]pyridazin-7-one: a bicyclic nucleoside with sugar residues in N and S con­formations, and its mol­ecular recognition
Source: Acta Crystallogr C Struct Chem. 2022 Jun 13;78(Pt 7):382–9. doi: 10.1107/S2053229622005964 (PMC9255914; doi:10.1107/S2053229622005964)
Supplement: Supplementary file 3 [file c-78-00382-sup3.pdf]

STRUCTURAL CHEMISTRY

Volume 78 (2022)

Supporting information for article:

The 2′-deoxyribofuranoside of 3-phenyltetrahydropyrimido[4,5-*c*]pyridazin-7-one: a bicyclic nucleoside with sugar residues in *N* and *S* conformations and its molecular recognition

Hui Mei, Simone Budow-Busse, Dasharath Kondhare, Henning Eickmeier, Hans Reuter and Frank Seela

Supporting Information

The 2’-deoxyribofuranoside of tetrahydro-3-phenyl-pyrimido[4,5-*c*]pyridazin-7-one: a bicyclic nucleoside with sugar residues in *N* and *S* conformation and its molecular recognition

Hui Mei[a], Simone Budow-Busse[a], Dasharath Kondhare[a], Henning Eickmeier[b], Hans Reuter[b] and Frank Seela[a,c*]

[a]*Laboratory of Bioorganic Chemistry and Chemical Biology, Center for Nanotechnology, Heisenbergstrasse 11, 48149 Münster, Germany*

[b]*Anorganische Chemie II, Institut für Chemie neuer Materialien, Universität Osnabrück, Barbarastrasse 7, 49069 Osnabrück, Germany*

[c]*Laboratorium für Organische und Bioorganische Chemie, Institut für Chemie neuer Materialien, Universität Osnabrück, Barbarastrasse 7, 49069 Osnabrück, Germany*

Table of Contents

Figure S1. Reverse alignment of the two conformers of the 3-methylpyrimido[4,5-*c*]pyridazine 2'-deoxyribonucleoside S3

Figure S2. Extended crystalline network of molecule **1** S3

Figure S3. Shape index surface of nucleoside **1** S4

Oligonucleotide syntheses and characterization S4-5

Figure S4. Melting curves of DNA duplexes S5

Literature S6

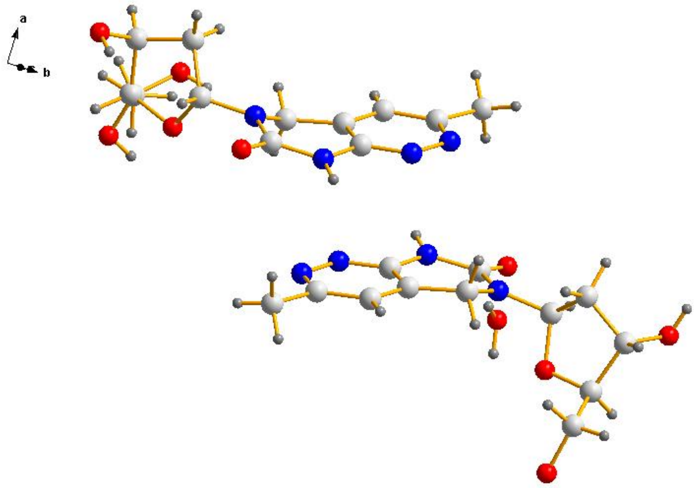

Figure S1. Reverse alignment of the two conformers of the 3-methylpyrimido[4,5-*c*]pyridazine 2'-deoxyribonucleoside (Loakes *et al.*, 2003).

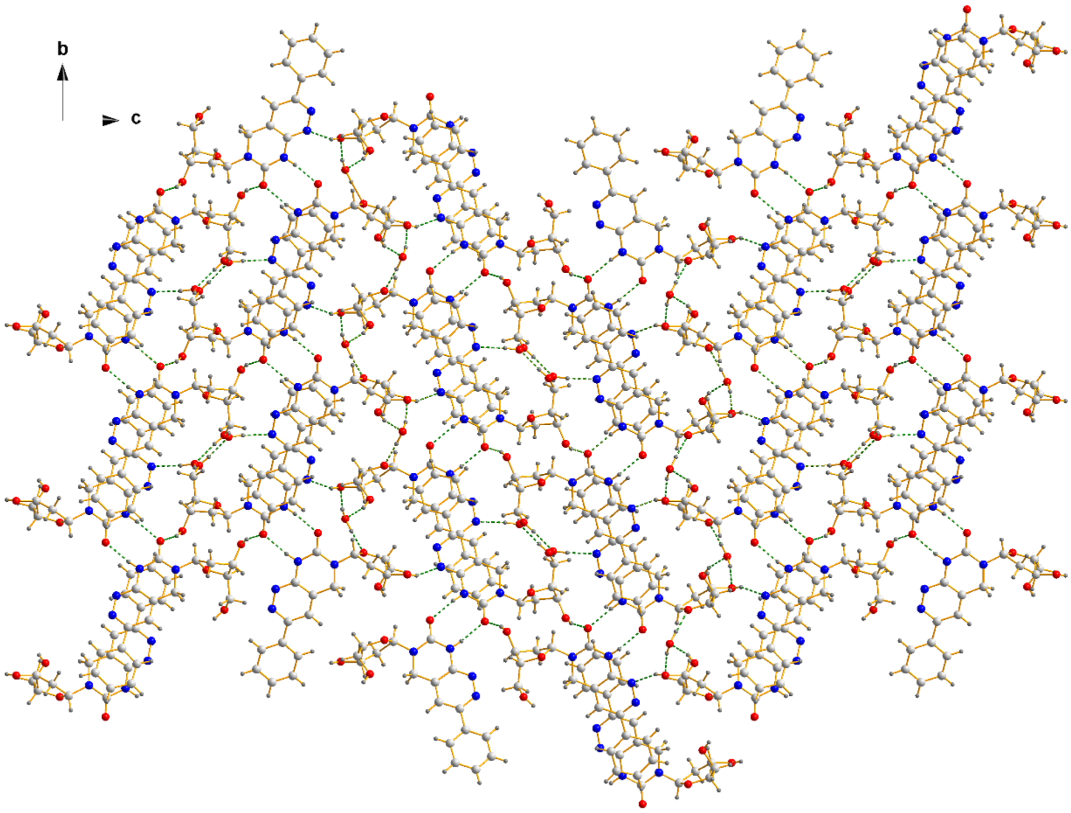

Figure S2. Extended crystalline network of molecule **1** and hydrogen bonding within the *bc* plane.

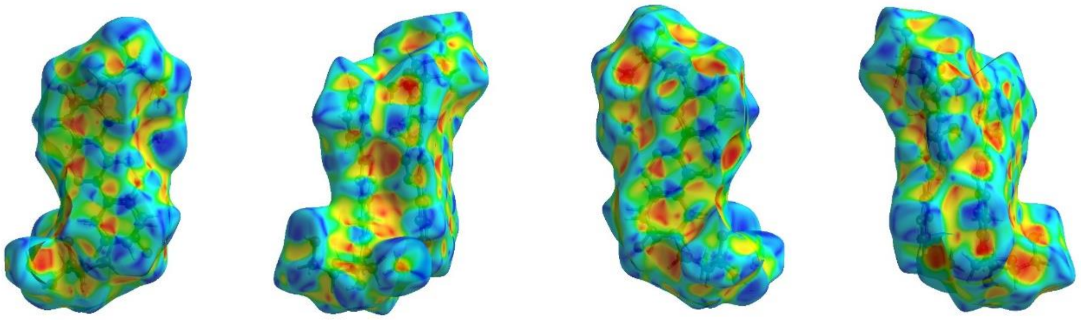

(*a*) (*b*) (*c*) (*d*)

Figure S3. Shape index (-1.0 to 1.0 Å) of the phenyl-pyrimido[4,5-*c*]pyridazine 2'-deoxyribonucleoside **1**. (*a*) Front view. (*b*), (*d*) Side viewa. (*d*) Back view.

Oligonucleotide syntheses, characterization and DNA hybridization experiments

Solid-phase oligonucleotide syntheses were performed with an ABI 392-08 synthesizer at 1 µmol scale (trityl-on mode) employing the phosphoramidites of nucleoside **1** (Mei *et al.*, 2014) and **4** (Seela & Becher, 2001) together with the phosphoramidites of the canonical nucleosides, giving an average coupling yield of over 95%. After cleavage from the solid support, the oligonucleotides were deprotected in 28% aqueous ammonia at 55°C for 12 h. The 4,4'-dimethoxytrityl containing oligonucleotides were purified by reversed-phase HPLC (RP-18) with a gradient system at 260 nm: (A) MeCN, (B) 0.1m $(Et_3NH)OAc$ (pH 7.0)/MeCN, 95:5; gradient I: 0–3 min 10–15% A in B, 3–15 min 15–50% A in B; flow rate 0.7 mL min^{-1}. The purified "trityl-on" oligonucleotides were treated with 2.5% $CHCl_2COOH$/ CH_2Cl_2 for 2 min at 8°C to remove the 4,4'-dimethoxytrityl residues. The detritylated oligomers were further purified by reversed phase HPLC with gradient II: 0–20 min 0–20% A in B; 20–25 min, 20% A in B; flow rate 0.7 mL min^{-1}. The oligonucleotides were desalted on a reversed-phase column (RP-18) by using water for the elution of salts, and the oligonucleotides were eluted with H_2O/MeOH (2:3). The oligonucleotides were lyophilized

with a SpeedVac evaporator to yield colorless solids, which were frozen at -24°C. The purity of all oligonucleotides was confirmed by RP-18 HPLC and MALDI-TOF mass spectrometry. The thermal melting curves of DNA oligonucleotide duplexes were measured with an Agilent Technologies Cary 100 Bio UV-vis spectrophotometer equipped with a thermoelectrical controller. The temperature was measured continuously in the reference cell with a Pt-100 resistor with a heating rate of 1.0 °C/min. All measurements were performed at 260 nm at a concentration of 2 µM + 2 µM single strand in 100 mM NaCl, 10 mM $MgCl_2$, and 10 mM Na-cacodylate (pH 7.0). T_m values were determined from the melting curves using the software *Meltwin*, version 3.0 (McDowell & Turner, 1996).

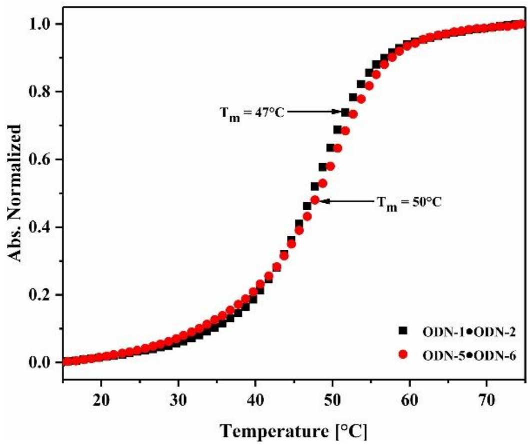

Figure S4. Melting curves of the DNA duplex ODN-**5**•ODN-**6** incorporating the modified base pair of **1** and **4** and the unmodified reference duplex ODN-**1**•ODN-**2**. All measurements were performed at 260 nm at a concentration of 2 µM + 2 µM single strand at a heating rate of 1.0 °C/min in 100 mM NaCl, 10 mM $MgCl_2$, and 10 mM Na-cacodylate (pH 7.0).

Literature

Loakes, D., Brown, D. M., Salisbury, S. A., McDougall, M. G., Neagu, C., Nampalli, S. & Kumar, S. (2003). *Helv. Chim. Acta*, **86**, 1193-1204.

McDowell, J. A. & Turner, D. H. (1996). *Biochemistry*, **35**, 14077-14089.

Mei, H., Ingale, S. A. & Seela, F. (2015). *Tetrahedron*, **71**, 6170-6175.

Seela, F. & Becher, G. (2001). *Nucleic Acids Res.* **29**, 2069-2078.

Computing details

Data collection: *APEX2* (Bruker, 2008); cell refinement: *SAINT* (Bruker, 2008); data reduction: *SAINT* (Bruker, 2008); program(s) used to solve structure: *SHELXTL2008/4* (Sheldrick, 2008); program(s) used to refine structure: *SHELXTL2008/4* (Sheldrick, 2008); molecular graphics: *SHELXTL2008/4* (Sheldrick, 2008) and *DIAMOND* (Brandenburg, 2005); software used to prepare material for publication: *SHELXTL2008/4* (Sheldrick, 2008) and *PLATON* (Spek, 2009).

6-(2-Deoxy-β-D-*erythro*-pentofuranosyl)-5,6,7,8-tetrahydro-3-phenylpyrimido[4,5-*c*]pyridazin-7-one monohydrate

Crystal data	
Chemical formula	$C_{17}H_{18}N_4O_4·H_2O$
M_r	360.37
Crystal system, space group	Orthorhombic, $P2_12_12_1$
Temperature (K)	100
a, *b*, *c* (Å)	7.2057 (3), 11.0792 (4), 41.2346 (16)
V (Å^3)	3291.9 (2)
Z	8
F(000)	1520
D_x (Mg m^{-3})	1.454
Radiation type	Mo *K*α
No. of reflections for cell measurement	9843
θ range (°) for cell measurement	2.7–24.5
μ (mm^{-1})	0.11
Crystal shape	Plate
Colour	Colourless
Crystal size (mm)	0.19 × 0.16 × 0.09
Data collection	
Diffractometer	Bruker *APEX*-II CCD
Radiation source	fine-focus sealed tube
Monochromator	Graphite
Scan method	φ and ω scans
Absorption correction	Multi-scan (*SADABS*; Bruker, 2008)
T_{min}, T_{max}	0.979, 0.991
No. of measured, independent and observed [> *I* > 2σ(*I*)] reflections	91832, 4528, 3773
R_{int}	0.118
θ values (°)	$θ_{max}$ = 28.0, $θ_{min}$ = 2.9
(sin θ/λ)$_{max}$ (Å^{-1})	0.661

Range of h, k, l	$h = -9 \rightarrow 9, k = -11 \rightarrow 14, l = -54 \rightarrow 54$
Refinement	
Refinement on	F^2
$R[F^2 > 2\sigma(F^2)], wR(F^2), S$	0.045, 0.096, 1.07
No. of reflections	4528
No. of parameters	473
No. of restraints	0
H-atom treatment	H atoms treated by a mixture of independent and constrained refinement
Weighting scheme	$w = 1/[\sigma^2(F_o^2) + (0.0414P)^2 + 1.187P]$ where $P = (F_o^2 + 2F_c^2)/3$
$(\Delta/\sigma)_{max}$	0.001
$\Delta\rho_{max}, \Delta\rho_{min}$ (e Å^{-3})	0.27, −0.31
Absolute structure	Established by known chemical absolute configuration

Special details

Geometry. All e.s.d.'s (except the e.s.d. in the dihedral angle between two l.s. planes) are estimated using the full covariance matrix. The cell e.s.d.'s are taken into account individually in the estimation of e.s.d.'s in distances, angles and torsion angles; correlations between e.s.d.'s in cell parameters are only used when they are defined by crystal symmetry. An approximate (isotropic) treatment of cell e.s.d.'s is used for estimating e.s.d.'s involving l.s. planes.

Refinement. Refinement of F^2 against ALL reflections. The weighted R-factor wR and goodness of fit S are based on F^2, conventional R-factors R are based on F, with F set to zero for negative F^2. The threshold expression of $F^2 > 2\sigma(F^2)$ is used only for calculating R-factors(gt) *etc.* and is not relevant to the choice of reflections for refinement. R-factors based on F^2 are statistically about twice as large as those based on F, and R-factors based on ALL data will be even larger.

Atomic coordinates and equivalent isotropic displacement parameters (Å^2)

	x	*y*	*z*	*U(eq)*
C11C	0.1272(3)	0.1163(2)	0.8616(1)	0.013(1)
C12C	0.1019(4)	0.0262(2)	0.8848(1)	0.017(1)
C13C	0.0356(4)	-0.0868(3)	0.8760(1)	0.023(1)
C14C	-0.0046(4)	-0.1125(3)	0.8441(1)	0.021(1)
C15C	0.0210(4)	-0.0242(3)	0.8206(1)	0.017(1)
C16C	0.0849(4)	0.0890(2)	0.8292(1)	0.015(1)
N11	0.3292(3)	0.4174(2)	0.8532(1)	0.012(1)
N12	0.2625(3)	0.3059(2)	0.8470(1)	0.012(1)

C13	0.1986(3)	0.2372(2)	0.8708(1)	0.012(1)
C14	0.2024(3)	0.2773(2)	0.9035(1)	0.011(1)
C14A	0.2721(3)	0.3879(2)	0.9100(1)	0.011(1)
C15	0.2906(4)	0.4370(2)	0.9437(1)	0.012(1)
N16	0.3299(3)	0.5667(2)	0.9442(1)	0.012(1)
C17	0.4030(3)	0.6273(2)	0.9184(1)	0.012(1)
O17	0.4676(3)	0.7303(2)	0.9207(1)	0.015(1)
N18	0.3986(3)	0.5716(2)	0.8888(1)	0.013(1)
C18A	0.3315(3)	0.4570(2)	0.8835(1)	0.010(1)
C11'	0.3522(3)	0.6207(2)	0.9762(1)	0.013(1)
C12'	0.1876(4)	0.6044(2)	0.9989(1)	0.013(1)
C13'	0.2784(4)	0.5957(2)	1.0322(1)	0.013(1)
O13'	0.3197(3)	0.7116(2)	1.0453(1)	0.021(1)
C14'	0.4620(4)	0.5328(2)	1.0246(1)	0.013(1)
O14'	0.5082(2)	0.5653(2)	0.9917(1)	0.015(1)
C15'	0.4452(4)	0.3978(2)	1.0280(1)	0.015(1)
O15'	0.6133(3)	0.3368(2)	1.0194(1)	0.019(1)
O100	0.4971(3)	0.1729(2)	0.9741(1)	0.019(1)
C21C	0.7559(4)	0.3511(2)	0.8909(1)	0.012(1)
C22C	0.8280(4)	0.4273(2)	0.8671(1)	0.015(1)
C23C	0.8992(4)	0.5400(2)	0.8752(1)	0.016(1)
C24C	0.8983(4)	0.5781(2)	0.9071(1)	0.017(1)
C25C	0.8263(4)	0.5037(2)	0.9309(1)	0.016(1)
C26C	0.7570(4)	0.3909(2)	0.9231(1)	0.014(1)
N21	0.5764(3)	0.0444(2)	0.9016(1)	0.015(1)
N22	0.6355(3)	0.1577(2)	0.9070(1)	0.014(1)
C23	0.6819(3)	0.2301(2)	0.8824(1)	0.011(1)
C24	0.6575(4)	0.1919(2)	0.8501(1)	0.012(1)
C24A	0.5947(3)	0.0782(2)	0.8441(1)	0.011(1)
C25	0.5541(4)	0.0310(2)	0.8109(1)	0.013(1)
N26	0.5008(3)	-0.0964(2)	0.8110(1)	0.013(1)
C27	0.4895(4)	-0.1679(2)	0.8379(1)	0.013(1)
O27	0.4581(3)	-0.2770(2)	0.8363(1)	0.018(1)
N28	0.5126(3)	-0.1135(2)	0.8672(1)	0.015(1)
C28A	0.5630(3)	0.0056(2)	0.8713(1)	0.012(1)
C21'	0.4844(4)	-0.1552(2)	0.7798(1)	0.014(1)
C22'	0.3656(4)	-0.0891(2)	0.7546(1)	0.015(1)
C23'	0.5028(4)	-0.0394(2)	0.7299(1)	0.012(1)
O23'	0.4186(3)	-0.0392(2)	0.6987(1)	0.016(1)
O24'	0.6678(3)	-0.1631(2)	0.7660(1)	0.017(1)
C24'	0.6665(4)	-0.1255(2)	0.7325(1)	0.015(1)
C25'	0.8534(4)	-0.0731(3)	0.7246(1)	0.020(1)
O25'	0.8859(3)	0.0315(2)	0.7437(1)	0.027(1)
O200	0.1440(3)	0.1422(2)	0.7058(1)	0.021(1)

Atomic displacement parameters (Å^2)

	U^{11}	U^{22}	U^{33}	U^{23}	U^{13}	U^{12}
C11C	0.0075(12)	0.0134(13)	0.0155(12)	-0.0015(11)	0.0009(9)	-0.0018(11)
C12C	0.0224(15)	0.0148(14)	0.0131(13)	-0.0004(11)	0.0015(11)	-0.0038(12)
C13C	0.0316(17)	0.0126(15)	0.0236(15)	0.0001(12)	0.0016(13)	-0.0063(13)
C14C	0.0205(14)	0.0135(14)	0.0289(15)	-0.0048(12)	0.0027(12)	-0.0057(13)
C15C	0.0151(13)	0.0203(15)	0.0146(12)	-0.0075(11)	0.0022(11)	0.0007(12)
C16C	0.0131(12)	0.0150(14)	0.0160(13)	0.0001(11)	0.0023(10)	-0.0014(12)
N11	0.0135(10)	0.0099(11)	0.0102(10)	0.0002(8)	-0.0004(8)	-0.0007(9)
N12	0.0145(11)	0.0108(11)	0.0104(10)	-0.0004(8)	-0.0002(9)	-0.0001(9)
C13	0.0084(12)	0.0113(13)	0.0135(12)	-0.0003(10)	-0.0013(10)	0.0001(10)
C14	0.0120(13)	0.0099(13)	0.0111(11)	0.0018(10)	0.0002(10)	0.0008(11)
C14A	0.0110(12)	0.0099(13)	0.0102(11)	0.0005(10)	0.0007(10)	0.0019(10)
C15	0.0193(13)	0.0084(13)	0.0083(11)	-0.0011(10)	0.0015(10)	-0.0016(11)
N16	0.0166(11)	0.0098(11)	0.0088(10)	-0.0017(8)	0.0011(9)	-0.0024(9)
C17	0.0129(12)	0.0102(13)	0.0099(12)	0.0007(10)	-0.0017(10)	0.0002(11)
O17	0.0229(10)	0.0117(9)	0.0101(8)	0.0019(7)	0.0000(8)	-0.0038(8)
N18	0.0185(11)	0.0116(11)	0.0076(10)	0.0016(8)	0.0017(9)	-0.0033(10)
C18A	0.0096(11)	0.0101(13)	0.0093(11)	0.0004(10)	0.0006(10)	-0.0001(10)
C11'	0.0176(13)	0.0106(13)	0.0091(11)	0.0003(10)	-0.0013(10)	-0.0021(11)
C12'	0.0146(13)	0.0125(13)	0.0107(11)	-0.0002(10)	-0.0008(10)	0.0028(11)
C13'	0.0151(13)	0.0111(13)	0.0111(12)	-0.0018(10)	0.0013(10)	-0.0009(11)
O13'	0.0249(11)	0.0156(10)	0.0203(10)	-0.0096(8)	0.0010(9)	0.0006(9)
C14'	0.0134(13)	0.0147(13)	0.0093(12)	-0.0009(10)	-0.0021(10)	-0.0010(11)
O14'	0.0139(9)	0.0212(10)	0.0081(8)	0.0021(7)	0.0002(7)	0.0012(8)
C15'	0.0147(13)	0.0152(14)	0.0140(12)	-0.0011(11)	0.0017(10)	0.0031(11)
O15'	0.0157(10)	0.0185(11)	0.0219(10)	-0.0070(8)	-0.0026(8)	0.0053(9)
O100	0.0151(9)	0.0221(11)	0.0170(9)	-0.0057(8)	-0.0005(8)	0.0012(9)
C21C	0.0101(12)	0.0104(13)	0.0136(12)	-0.0015(10)	-0.0023(10)	0.0011(10)
C22C	0.0163(13)	0.0140(14)	0.0122(12)	0.0004(10)	-0.0019(10)	0.0017(11)
C23C	0.0147(13)	0.0132(14)	0.0197(14)	0.0024(11)	0.0001(11)	0.0003(12)
C24C	0.0150(13)	0.0101(14)	0.0234(14)	-0.0046(11)	0.0013(11)	-0.0015(11)
C25C	0.0143(13)	0.0157(14)	0.0148(12)	-0.0046(11)	0.0005(11)	0.0009(12)
C26C	0.0114(12)	0.0150(13)	0.0136(12)	-0.0021(11)	0.0003(10)	-0.0003(11)
N21	0.0187(12)	0.0111(11)	0.0123(10)	-0.0007(9)	-0.0020(9)	-0.0004(10)
N22	0.0154(11)	0.0125(12)	0.0132(10)	-0.0019(9)	-0.0013(9)	0.0012(10)
C23	0.0096(11)	0.0118(13)	0.0109(11)	0.0002(10)	-0.0012(10)	0.0024(11)

C24	0.0132(13)	0.0111(13)	0.0113(12)	0.0017(10)	0.0022(10)	0.0013(11)
C24A	0.0105(12)	0.0119(13)	0.0107(11)	0.0007(10)	0.0010(10)	0.0002(11)
C25	0.0176(13)	0.0088(13)	0.0105(12)	0.0017(10)	-0.0016(10)	-0.0019(11)
N26	0.0188(11)	0.0094(11)	0.0082(9)	-0.0008(8)	0.0005(9)	-0.0021(10)
C27	0.0143(13)	0.0127(14)	0.0108(12)	-0.0004(10)	0.0022(10)	0.0001(11)
O27	0.0314(12)	0.0104(10)	0.0114(9)	0.0011(8)	0.0004(8)	-0.0039(9)
N28	0.0261(12)	0.0110(11)	0.0077(10)	0.0028(9)	0.0012(9)	-0.0024(10)
C28A	0.0137(12)	0.0090(13)	0.0120(12)	-0.0002(10)	0.0002(10)	0.0008(10)
C21'	0.0170(13)	0.0136(14)	0.0110(12)	-0.0013(10)	0.0009(10)	-0.0012(12)
C22'	0.0135(13)	0.0199(15)	0.0103(12)	-0.0003(11)	0.0000(10)	-0.0014(11)
C23'	0.0164(13)	0.0119(13)	0.0070(11)	-0.0016(10)	-0.0003(10)	-0.0036(11)
O23'	0.0158(9)	0.0239(11)	0.0071(8)	0.0016(8)	-0.0005(7)	0.0016(9)
O24'	0.0160(9)	0.0210(10)	0.0108(9)	0.0028(8)	0.0011(7)	0.0066(9)
C24'	0.0186(13)	0.0166(14)	0.0069(11)	-0.0008(10)	-0.0004(10)	0.0015(12)
C25'	0.0156(14)	0.0306(17)	0.0128(13)	-0.0050(12)	0.0020(11)	-0.0031(13)
O25'	0.0251(12)	0.0359(13)	0.0194(10)	-0.0068(9)	0.0046(9)	-0.0166(10)
O200	0.0159(10)	0.0172(10)	0.0264(10)	-0.0024(8)	-0.0005(8)	-0.0021(9)

Geometric parameters (Å, º)

C11C—C12C	1.396 (4)	C21C—C22C	1.393 (4)
C11C—C16C	1.401 (4)	C21C—C26C	1.400 (3)
C11C—C13	1.483 (4)	C21C—C23	1.486 (4)
C12C—C13C	1.387 (4)	C22C—C23C	1.391 (4)
C12C—H12C	0.9500	C22C—H22C	0.9500
C13C—C14C	1.380 (4)	C23C—C24C	1.384 (4)
C13C—H13C	0.9500	C23C—H23C	0.9500
C14C—C15C	1.389 (4)	C24C—C25C	1.383 (4)
C14C—H14C	0.9500	C24C—H24C	0.9500
C15C—C16C	1.383 (4)	C25C—C26C	1.384 (4)
C15C—H15C	0.9500	C25C—H25C	0.9500
C16C—H16C	0.9500	C26C—H26C	0.9500
N11—C18A	1.327 (3)	N21—C28A	1.322 (3)
N11—N12	1.349 (3)	N21—N22	1.344 (3)
N12—C13	1.328 (3)	N22—C23	1.337 (3)

C13—C14	1.419 (3)	C23—C24	1.409 (3)
C14—C14A	1.351 (4)	C24—C24A	1.360 (4)
C14—H14	0.9500	C24—H24	0.9500
C14A—C18A	1.401 (3)	C24A—C28A	1.399 (3)
C14A—C15	1.499 (3)	C24A—C25	1.495 (3)
C15—N16	1.465 (3)	C25—N26	1.463 (3)
C15—H15E	0.9900	C25—H25D	0.9900
C15—H15D	0.9900	C25—H25E	0.9900
N16—C17	1.365 (3)	N26—C27	1.367 (3)
N16—C11'	1.457 (3)	N26—C21'	1.448 (3)
C17—O17	1.237 (3)	C27—O27	1.232 (3)
C17—N18	1.368 (3)	C27—N28	1.358 (3)
N18—C18A	1.375 (3)	N28—C28A	1.379 (3)
N18—H18N	0.8800	N28—H28N	0.8800
C11'—O14'	1.432 (3)	C21'—O24'	1.441 (3)
C11'—C12'	1.523 (3)	C21'—C22'	1.533 (4)
C11'—H11'	1.0000	C21'—H21'	1.0000
C12'—C13'	1.522 (3)	C22'—C23'	1.522 (3)
C12'—H12A	0.9900	C22'—H22A	0.9900
C12'—H12B	0.9900	C22'—H22B	0.9900
C13'—O13'	1.425 (3)	C23'—O23'	1.423 (3)
C13'—C14'	1.528 (4)	C23'—C24'	1.519 (4)
C13'—H13'	1.0000	C23'—H23'	1.0000
O13'—H13O	0.8400	O23'—H23O	0.8400
C14'—O14'	1.440 (3)	O24'—C24'	1.441 (3)
C14'—C15'	1.508 (4)	C24'—C25'	1.503 (4)
C14'—H14'	1.0000	C24'—H24'	1.0000
C15'—O15'	1.431 (3)	C25'—O25'	1.422 (3)
C15'—H15A	0.9900	C25'—H25A	0.9900
C15'—H15B	0.9900	C25'—H25B	0.9900
O15'—H15O	0.8400	O25'—H25O	0.8400
O100—H101	0.9600	O200—H201	0.9600
O100—H100	0.9602	O200—H200	0.9601
C12C—C11C—C16C	118.0 (2)	C22C—C21C—C26C	118.4 (2)

C12C—C11C—C13	121.0 (2)	C22C—C21C—C23	120.8 (2)
C16C—C11C—C13	121.0 (2)	C26C—C21C—C23	120.7 (2)
C13C—C12C—C11C	120.9 (2)	C23C—C22C—C21C	120.9 (2)
C13C—C12C—H12C	119.6	C23C—C22C—H22C	119.6
C11C—C12C—H12C	119.6	C21C—C22C—H22C	119.6
C14C—C13C—C12C	120.5 (3)	C24C—C23C—C22C	120.0 (2)
C14C—C13C—H13C	119.8	C24C—C23C—H23C	120.0
C12C—C13C—H13C	119.8	C22C—C23C—H23C	120.0
C13C—C14C—C15C	119.5 (3)	C25C—C24C—C23C	119.7 (2)
C13C—C14C—H14C	120.3	C25C—C24C—H24C	120.1
C15C—C14C—H14C	120.3	C23C—C24C—H24C	120.1
C16C—C15C—C14C	120.3 (2)	C24C—C25C—C26C	120.6 (2)
C16C—C15C—H15C	119.8	C24C—C25C—H25C	119.7
C14C—C15C—H15C	119.8	C26C—C25C—H25C	119.7
C15C—C16C—C11C	120.8 (2)	C25C—C26C—C21C	120.5 (2)
C15C—C16C—H16C	119.6	C25C—C26C—H26C	119.8
C11C—C16C—H16C	119.6	C21C—C26C—H26C	119.8
C18A—N11—N12	119.1 (2)	C28A—N21—N22	118.9 (2)
C13—N12—N11	120.5 (2)	C23—N22—N21	120.9 (2)
N12—C13—C14	121.2 (2)	N22—C23—C24	120.4 (2)
N12—C13—C11C	116.5 (2)	N22—C23—C21C	116.8 (2)
C14—C13—C11C	122.2 (2)	C24—C23—C21C	122.7 (2)
C14A—C14—C13	118.6 (2)	C24A—C24—C23	119.3 (2)
C14A—C14—H14	120.7	C24A—C24—H24	120.4
C13—C14—H14	120.7	C23—C24—H24	120.4
C14—C14A—C18A	117.1 (2)	C24—C24A—C28A	116.3 (2)
C14—C14A—C15	123.1 (2)	C24—C24A—C25	123.6 (2)
C18A—C14A—C15	119.8 (2)	C28A—C24A—C25	120.1 (2)
N16—C15—C14A	112.7 (2)	N26—C25—C24A	112.8 (2)
N16—C15—H15E	109.1	N26—C25—H25D	109.0
C14A—C15—H15E	109.1	C24A—C25—H25D	109.0
N16—C15—H15D	109.1	N26—C25—H25E	109.0
C14A—C15—H15D	109.1	C24A—C25—H25E	109.0
H15E—C15—H15D	107.8	H25D—C25—H25E	107.8

C17—N16—C11'	117.5 (2)	C27—N26—C21'	117.2 (2)
C17—N16—C15	123.1 (2)	C27—N26—C25	125.2 (2)
C11'—N16—C15	115.87 (19)	C21'—N26—C25	117.0 (2)
O17—C17—N16	122.6 (2)	O27—C27—N28	120.5 (2)
O17—C17—N18	119.7 (2)	O27—C27—N26	122.3 (2)
N16—C17—N18	117.7 (2)	N28—C27—N26	117.2 (2)
C17—N18—C18A	124.5 (2)	C27—N28—C28A	124.5 (2)
C17—N18—H18N	117.8	C27—N28—H28N	117.7
C18A—N18—H18N	117.8	C28A—N28—H28N	117.7
N11—C18A—N18	117.3 (2)	N21—C28A—N28	116.6 (2)
N11—C18A—C14A	123.4 (2)	N21—C28A—C24A	123.8 (2)
N18—C18A—C14A	119.3 (2)	N28—C28A—C24A	119.6 (2)
O14'—C11'—N16	108.5 (2)	O24'—C21'—N26	107.7 (2)
O14'—C11'—C12'	106.55 (19)	O24'—C21'—C22'	105.87 (19)
N16—C11'—C12'	115.0 (2)	N26—C21'—C22'	115.7 (2)
O14'—C11'—H11'	108.9	O24'—C21'—H21'	109.1
N16—C11'—H11'	108.9	N26—C21'—H21'	109.1
C12'—C11'—H11'	108.9	C22'—C21'—H21'	109.1
C13'—C12'—C11'	103.2 (2)	C23'—C22'—C21'	105.2 (2)
C13'—C12'—H12A	111.1	C23'—C22'—H22A	110.7
C11'—C12'—H12A	111.1	C21'—C22'—H22A	110.7
C13'—C12'—H12B	111.1	C23'—C22'—H22B	110.7
C11'—C12'—H12B	111.1	C21'—C22'—H22B	110.7
H12A—C12'—H12B	109.1	H22A—C22'—H22B	108.8
O13'—C13'—C12'	112.0 (2)	O23'—C23'—C24'	113.4 (2)
O13'—C13'—C14'	108.0 (2)	O23'—C23'—C22'	109.1 (2)
C12'—C13'—C14'	102.44 (19)	C24'—C23'—C22'	103.3 (2)
O13'—C13'—H13'	111.3	O23'—C23'—H23'	110.3
C12'—C13'—H13'	111.3	C24'—C23'—H23'	110.3
C14'—C13'—H13'	111.3	C22'—C23'—H23'	110.3
C13'—O13'—H13O	109.5	C23'—O23'—H23O	109.5
O14'—C14'—C15'	110.8 (2)	C24'—O24'—C21'	110.73 (18)
O14'—C14'—C13'	106.2 (2)	O24'—C24'—C25'	108.3 (2)
C15'—C14'—C13'	111.3 (2)	O24'—C24'—C23'	104.78 (19)

O14'—C14'—H14'	109.5	C25'—C24'—C23'	115.9 (2)
C15'—C14'—H14'	109.5	O24'—C24'—H24'	109.2
C13'—C14'—H14'	109.5	C25'—C24'—H24'	109.2
C11'—O14'—C14'	110.27 (18)	C23'—C24'—H24'	109.2
O15'—C15'—C14'	112.2 (2)	O25'—C25'—C24'	109.9 (2)
O15'—C15'—H15A	109.2	O25'—C25'—H25A	109.7
C14'—C15'—H15A	109.2	C24'—C25'—H25A	109.7
O15'—C15'—H15B	109.2	O25'—C25'—H25B	109.7
C14'—C15'—H15B	109.2	C24'—C25'—H25B	109.7
H15A—C15'—H15B	107.9	H25A—C25'—H25B	108.2
C15'—O15'—H15O	109.5	C25'—O25'—H25O	109.5
H101—O100—H100	105.0	H201—O200—H200	105.0

C16C—C11C—C12C—C13C	−0.4 (4)	C26C—C21C—C22C—C23C	0.0 (4)
C13—C11C—C12C—C13C	−179.8 (3)	C23—C21C—C22C—C23C	−179.3 (2)
C11C—C12C—C13C—C14C	0.7 (5)	C21C—C22C—C23C—C24C	−0.3 (4)
C12C—C13C—C14C—C15C	−0.2 (5)	C22C—C23C—C24C—C25C	0.0 (4)
C13C—C14C—C15C—C16C	−0.5 (4)	C23C—C24C—C25C—C26C	0.7 (4)
C14C—C15C—C16C—C11C	0.8 (4)	C24C—C25C—C26C—C21C	−1.0 (4)
C12C—C11C—C16C—C15C	−0.4 (4)	C22C—C21C—C26C—C25C	0.6 (4)
C13—C11C—C16C—C15C	179.1 (2)	C23—C21C—C26C—C25C	179.9 (2)
C18A—N11—N12—C13	0.6 (3)	C28A—N21—N22—C23	−0.6 (4)
N11—N12—C13—C14	−1.5 (4)	N21—N22—C23—C24	4.4 (4)
N11—N12—C13—C11C	179.7 (2)	N21—N22—C23—C21C	−176.5 (2)
C12C—C11C—C13—N12	163.4 (2)	C22C—C21C—C23—N22	172.4 (2)
C16C—C11C—C13—N12	−16.1 (4)	C26C—C21C—C23—N22	−6.8 (4)
C12C—C11C—C13—C14	−15.4 (4)	C22C—C21C—C23—C24	−8.5 (4)
C16C—C11C—C13—C14	165.1 (2)	C26C—C21C—C23—C24	172.2 (2)
N12—C13—C14—C14A	0.2 (4)	N22—C23—C24—C24A	−3.2 (4)
C11C—C13—C14—C14A	179.0 (2)	C21C—C23—C24—C24A	177.8 (2)
C13—C14—C14A—C18A	1.8 (4)	C23—C24—C24A—C28A	−1.6 (4)
C13—C14—C14A—C15	−177.2 (2)	C23—C24—C24A—C25	176.5 (2)
C14—C14A—C15—N16	−167.1 (2)	C24—C24A—C25—N26	176.0 (2)
C18A—C14A—C15—N16	13.9 (3)	C28A—C24A—C25—N26	−6.1 (3)
C14A—C15—N16—C17	−20.9 (3)	C24A—C25—N26—C27	−0.6 (4)

C14A—C15—N16—C11'	−179.2 (2)	C24A—C25—N26—C21'	−171.4 (2)
C11'—N16—C17—O17	−8.2 (4)	C21'—N26—C27—O27	−3.3 (4)
C15—N16—C17—O17	−166.1 (2)	C25—N26—C27—O27	−174.1 (3)
C11'—N16—C17—N18	173.7 (2)	C21'—N26—C27—N28	177.3 (2)
C15—N16—C17—N18	15.7 (4)	C25—N26—C27—N28	6.5 (4)
O17—C17—N18—C18A	179.4 (2)	O27—C27—N28—C28A	174.4 (3)
N16—C17—N18—C18A	−2.4 (4)	N26—C27—N28—C28A	−6.2 (4)
N12—N11—C18A—N18	−179.3 (2)	N22—N21—C28A—N28	176.2 (2)
N12—N11—C18A—C14A	1.6 (4)	N22—N21—C28A—C24A	−4.5 (4)
C17—N18—C18A—N11	177.2 (2)	C27—N28—C28A—N21	179.0 (2)
C17—N18—C18A—C14A	−3.7 (4)	C27—N28—C28A—C24A	−0.3 (4)
C14—C14A—C18A—N11	−2.8 (4)	C24—C24A—C28A—N21	5.5 (4)
C15—C14A—C18A—N11	176.2 (2)	C25—C24A—C28A—N21	−172.6 (2)
C14—C14A—C18A—N18	178.1 (2)	C24—C24A—C28A—N28	−175.2 (2)
C15—C14A—C18A—N18	−2.9 (4)	C25—C24A—C28A—N28	6.7 (4)
C17—N16—C11'—O14'	−97.5 (3)	C27—N26—C21'—O24'	−103.8 (3)
C15—N16—C11'—O14'	62.0 (3)	C25—N26—C21'—O24'	67.8 (3)
C17—N16—C11'—C12'	143.3 (2)	C27—N26—C21'—C22'	138.1 (2)
C15—N16—C11'—C12'	−57.1 (3)	C25—N26—C21'—C22'	−50.3 (3)
O14'—C11'—C12'—C13'	26.9 (3)	O24'—C21'—C22'—C23'	−11.4 (3)
N16—C11'—C12'—C13'	147.1 (2)	N26—C21'—C22'—C23'	107.7 (2)
C11'—C12'—C13'—O13'	82.3 (2)	C21'—C22'—C23'—O23'	147.3 (2)
C11'—C12'—C13'—C14'	−33.2 (3)	C21'—C22'—C23'—C24'	26.3 (3)
O13'—C13'—C14'—O14'	−89.8 (2)	N26—C21'—O24'—C24'	−133.6 (2)
C12'—C13'—C14'—O14'	28.6 (2)	C22'—C21'—O24'—C24'	−9.2 (3)
O13'—C13'—C14'—C15'	149.5 (2)	C21'—O24'—C24'—C25'	150.6 (2)
C12'—C13'—C14'—C15'	−92.1 (2)	C21'—O24'—C24'—C23'	26.2 (3)
N16—C11'—O14'—C14'	−133.5 (2)	O23'—C23'—C24'—O24'	−149.9 (2)
C12'—C11'—O14'—C14'	−9.2 (3)	C22'—C23'—C24'—O24'	−31.9 (2)
C15'—C14'—O14'—C11'	108.6 (2)	O23'—C23'—C24'—C25'	90.7 (3)
C13'—C14'—O14'—C11'	−12.4 (3)	C22'—C23'—C24'—C25'	−151.3 (2)
O14'—C14'—C15'—O15'	59.2 (3)	O24'—C24'—C25'—O25'	−63.0 (3)
C13'—C14'—C15'—O15'	177.20 (19)	C23'—C24'—C25'—O25'	54.4 (3)

Hydrogen-bond geometry (Å, º)

D—H···*A*	*D*—H	H···*A*	*D*···*A*	*D*—H···*A*
N18—H18N···O27^{i}	0.88	1.92	2.773 (3)	161.6
O13'—H13O···O17^{ii}	0.84	2.15	2.968 (3)	166.0
O15'—H15O···O100	0.84	1.91	2.736 (2)	169.3
O100—H101···O15'^{iii}	0.96	1.86	2.781 (3)	159.7
O100—H100···N22	0.96	1.99	2.946 (3)	175.0
N28—H28N···O17^{iv}	0.88	1.95	2.823 (3)	173.2
O23'—H23O···N11^{v}	0.84	2.05	2.846 (3)	158.3
O25'—H25O···O200^{vi}	0.84	1.90	2.721 (3)	164.3
O200—H201···O24'^{vii}	0.96	1.84	2.801 (3)	175.2
O200—H200···O23'	0.96	1.89	2.836 (3)	168.8

Symmetry code(s): (i) *x*, *y*+1, *z*; (ii) *x*−1/2, −*y*+3/2, −*z*+2; (iii) *x*−1/2, −*y*+1/2, −*z*+2; (iv) *x*, *y*−1, *z*; (v) −*x*+1, *y*−1/2, −*z*+3/2; (vi) *x*+1, *y*, *z*; (vii) −*x*+1, *y*+1/2, −*z*+3/2.
